# Growth Hormone Supplementation May Not Improve Live Birth Rate in Poor Responders

**DOI:** 10.3389/fendo.2020.00001

**Published:** 2020-01-23

**Authors:** Jinliang Zhu, Ying Wang, Lixue Chen, Ping Liu, Rong Li, Jie Qiao

**Affiliations:** ^1^Department of Obstetrics and Gynecology, Center for Reproductive Medicine, Peking University Third Hospital, Beijing, China; ^2^National Clinical Research Center for Obstetrics and Gynecology, Beijing, China

**Keywords:** poor ovarian responder, growth hormone, live birth rate, ovarian stimulation protocol, *in vitro* fertilization

## Abstract

**Backgrounds:** Growth hormone (GH) was used for many years to increase ovarian response in poor ovarian responders (PORs). Although meta-analysis suggested that GH therapy improve early clinical outcomes, the benefit of GH usage on chance of live birth was still widely debated. This study was to determine whether or not GH supplementation influences the live birth rate (LBR).

**Methods:** A total of 3,080 expected PORs receiving and not receiving (control) GH adjuvant therapy at Peking University Third Hospital from January 2017 to March 2018 were retrospectively analyzed. The basal characteristics of patients were compared using analysis of variance (continuous variables) and categorical variables were evaluated with a chi-square test. Logistic regression analyses were used to evaluate potential associations of LBR with GH treatment while adjusting other confounding factors.

**Results:** No statistically significant differences existed in miscarriage rate (5.3 vs. 12.5%; *p* = 0.076) and LBR (37.7 vs. 34.5%; *p* = 0.426) in young expected PORs (< 35 years of age). Moreover, no significant differences existed in the miscarriage rate (25.6 vs. 23.3%; *p* = 0.681), and LBR (17.8 vs. 17.9%; *p* = 0.977) in the old expected PORs (≥35 years of age). Logistic regression suggested that GH adjuvant therapy did not improve the LBR in young (OR, 1.27; 95% CI, 0.88–1.85; *p* = 0.203) and elderly expected PORs (OR, 1.20; 95% CI, 0.82–1.76; *p* = 0.342), while GH was not associated with risk of miscarriage in young (OR, 0.37; 95% CI, 0.11–1.24; *p* = 0.108) and elderly expected PORs (OR, 0.91; 95% CI, 0.43–1.93; *p* = 0.813). In subgroup analysis, GH treatment significantly increased the day 3 embryos available rate in the subgroup of young PORs with the long down-regulation (63.11 vs. 49.35%; *p* = 0.004), while significantly reduced the risk of miscarriage in the subgroup of young PORs with GnRH antagonist protocol (0.00 vs. 12. %; *p* = 0.023). There was no significant difference for LBR in PORs with GnRH antagonist (<35 years [35.19 vs. 28.45%; *p* = 0.183]; ≥35 years [12.96 vs. 14.03%; *p* = 0.707]), GnRH-a long (<35 years [33.33 vs. 36.99%; *p* = 0.597]; ≥35 years [17.44 vs. 20.28%; *p* = 0.574]) and long down-regulation (<35 years [58.82 vs. 41.90%; *p* = 0.193]; ≥35 years [43.33 vs. 25.30%; *p* = 0.065]).

**Conclusions:** Growth hormone treatment may not improve live birth rate in expected poor responders.

## Introduction

Controlled ovarian hyperstimulation (COH) was used to achieve multi-follicular development. A poor response to ovarian stimulation is estimated to occur in 5.6–35.1% of patients (1–6), depending on the definition of poor ovarian responder (POR). A poor response to ovarian stimulation is not common, and presents a significant therapeutic challenge. Many approaches have been proposed to improve clinical outcomes of PORs, including a modified ovarian stimulation protocol (7, 8), androgen treatment (9, 10), and a stem cell ovarian transplant (11).

The reasonable use of growth hormone (GH) in PORs is based on the requirement for follicular growth in animal studies (12, 13). Then, GH was reported to increase the density of granulosa follicle-stimulating hormone (FSH) and luteinizing hormone receptors in older PORs (14) and enhance oocyte mitochondrial activity in older women (15). GH has been used for many years to increase the ovarian response in clinical practice (16, 17). A number of studies have suggested that GH adjuvant therapy increases the number of retrieved oocytes, mature oocytes, and good quality embryos in POR cohorts (18–23). Moreover, a systematic review, including randomized controlled trials and single center retrospective studies, suggested that the addition of GH in ovarian stimulation increased the probability of clinical pregnancies and live births in PORs (24–26). However, a recent updated meta-analysis, including the largest multi-centered randomized controlled trial (RCT) conducted by the LIGHT investigators failed to find a significant difference in the live birth rate (LBR) after GH adjuvant therapy in PORs (27).

The discrepant finding may be related to the lack of a clear definition of POR and participation heterogeneity. A systematic review of 47 RCTs used 41 different definitions of a POR (28). To standardize the POR, the POSEIDON group proposed a more detailed definition of a POR (29), compared to the “Bologna criteria.” The POSEIDON group divided patients into unexpected and expected PORs based on the antral follicle count (AFC) and/or the anti-Mullerian hormone (AMH) level. As underlying etiologies of PORs were different in young and old sub-PORs, it is important that maternal age be taken into consideration in the POSEIDON classification. In view of the advantages of the POSEIDON classification, we set out to investigate the efficacy of GH adjuvant therapy in expected PORs based on POSEIDON classification. We hypothesized that GH treatment might benefit for sub-PORs in terms of increased LBR based on POSEIDON.

## Methods

### Participants

A total of 3,080 expected PORs undergoing the first fresh *in vitro* fertilization (IVF)/ intracytoplasmic sperm injection (ICSI) cycles from January 2017 to June 2018 at Peking University Third Hospital were included. The inclusion criterion were based on the definition of expected PORs released by POSEIDON in 2016 (29), as follows: POSEIDON group 3 (PG3) includes patients <35 years of age with poor pre-stimulation ovarian reserve parameters (AFC < 5 and/or AMH < 1.2 ng/mL); and POSEIDON group 4 (PG4) includes patients ≥35 years of age with poor pre-stimulation ovarian reserve parameters (AFC < 5 and/or AMH < 1.2 ng/mL). The exclusion criteria were as follows: (1) azoospermia or severe oligospermia; and (2) abnormal karyotyping. In the PG3 group, 271 and 1021 women received or did not (control) receive GH adjuvant therapy, respectively. In the PG4 group, 557 and 1,231 women received and did not receive (control) GH adjuvant therapy, respectively.

### Protocols

COH was achieved in those patients by using either recombinant FSH (rFSH) or human menopausal gonadotropin (HMG) in various flexible protocols. Each COH protocol with and without GH was used in our center based on physician experience. In the long gonadotrophin-releasing hormone agonist (GnRH-a) protocol, patients were administered a 0.1 mg triptorelin daily injection for 14 days or a single 1.3/1.8 mg triptorelin injection during the midterm luteal phase of the previous menstrual period followed by recombinant FSH (Gonal-f; Merck Serono, Geneva, Switzerland and Purigon, Organon, P.O. Box 20 OssNL5340BH, Netherlands) or hMG (Livzon Pharmaceutical Group, Zhuhai, China). In the long down-regulation (LDR), patients underwent pituitary down-regulation by 3.75 mg of triptorelin acetate or leuprorelin acetate on the first day of the cycle followed by rFSH or HMG 28–35 days later. In the flare cycle, patient received GnRH-a from day 2nd of the menstrual cycle onward then rFSH or HMG were given in the 3rd day. In the antagonist protocol, patients started with rFSH treatment on the 2nd day of the cycle by once-daily injection. Follicle development was monitored by ultrasound. After 5 days of this treatment, the antagonist (cetrorelix acetate or ganirelix acetate) was administered daily. The rFSH dose was adjusted according to individual ovarian response, which was assessed by daily ultrasound examination. The antagonist continued up to and including human chorionic gonadotropin (hCG) day. In all treatment protocols, when at least 2 leading follicles reached 18 mm in size, ovulation was induced by administering 250 μg of r-hCG (MerckSerono S.p.A), and ovum collection was performed between 34 and 38 h later. Progesterone gel (90 mg) was inserted into the vagina daily for 14, 21, and 30 days after fresh embryo transfer. Patients in the GH group received 4 IU/d of GH (Saizen; Merck Serono, Geneva, Switzerland), beginning on the initial day of gonadotrophin until the day of hCG injection. Criteria for treating PORs with GH was mainly based on physician experience, and affordability was one of factors in assigning GH treatment to PORs.

### Outcomes Measured

The main outcomes of the study were as follows: number of oocytes collected; day 3 embryos available rate; implantation rate (IR); clinical pregnancy rate (CPR) per transfer cycle; miscarriage rate (MR); and LBR per transfer cycle. An embryo was defined as an available day 3 embryo if the embryo had ≥5 cells and contained <20% anucleated fragments. The IR was calculated as the ratio of the number of gestational sacs to the number of embryos transferred. Clinical pregnancy was diagnosed when a gestational sac was detected by transvaginal ultrasound scan 4 weeks after embryo transfer. Live birth was defined as the birth in which at least one fetus was live born after 28 weeks gestation.

### Statistical Analysis

All statistical analyses were performed with the Statistical Package for the Social Sciences software (SPSS, version 17.0; SPSS, Inc., Chicago, IL, USA). The basal characteristics of patients were compared using analysis of variance (continuous variables) and categorical variables were evaluated with a chi-square test. Logistical regression analyses were used to evaluate potential associations of LBR and miscarriage rate with GH treatment while adjusting other confounding factors, including maternal age/body mass index (BMI), infertility duration, the type of infertility, female parity, main infertility causes, AMH, AFC, ovarian stimulation protocol, the number of collected oocytes, the number of transferred embryos, and transfer stage (day 3 vs. day 5). All tests were two-sided, a *P* < 0.05 was considered to be statistically significant.

### Ethical Approval

This retrospective cohort study was approved by the Ethics Committee of Reproductive Medicine, Peking University Third Hospital on 16 AUG 2019, the reference number was 2019SZ-062.

## Results

A total of 3080 women were recruited to the study. Eight hundred twenty-eight women underwent IVF/ICSI fresh cycles with GH co-treatment; 2252 women who did not receive GH co-treatment comprised the control group. There was no significant difference in the LBR between the two groups (25.05 vs. 25.93%; *p* = 0.698). Moreover, the LBR was similar between the GH and control groups with single embryo transfer stratified by days 3 and 5 transfers (D3, 15.84 vs. 12.23%; p = 0.373 and D5, 10.71 vs. 16.83%; *p* = 0.562). The trend was the same in double embryo transfers (Table 1). Consequently, we divided the patients into PG3 and PG4 for comparison.

**Table 1:** Live birth rate in single and double embryo transfer stratified by days 3 and 5 transfers.

**Live birth rate**	**All**				
	**GH**		**CN**		***P*-value**
Day 3 ET	122/503	(24.25%)	353/1,388	(25.43%)	0.602
SET	16/101	(15.84%)	28/229	(12.23%)	0.373
DET	106/402	(26.37%)	325/1,159	(28.04%)	0.518
Day 5 ET	4/29	(13.79%)	17/101	(16.83%)	1.000
SET	3/28	(10.71%)	17/101	(16.83%)	0.562
DET	1/1	(100%)	—		—

*GH, growth hormone; CN, control; ET, embryo transfer; SET, single embryo transfer; DET, double embryo transfer*.

### Clinical Outcomes in the PG 3

A total of 1,292 women were recruited to the PG3 group. In this group, 271 women were assigned to the GH adjuvant therapy group and 1,021 women were assigned to the control group. The baseline characteristics of patients and ovarian stimulation are shown in Table 2. There was no statistically significant difference in the baseline characteristics of patients and ovarian stimulation between GH treatment and control groups, except for duration of infertility (3.67 ± 2.52 vs. 3.29 ± 2.48; *p* = 0.030) and the protocol used for ovarian stimulation (*p* < 0.001). The number of cycles canceled before oocyte retrieval (2.58 vs. 2.15%; *p* = 0.672) was not significantly different between the two groups.

**Table 2:** Basal characteristics of patients and ovarian stimulation.

	**Maternal age** ** <35 years**					**Maternal age** **≥35 years**				
	**GH**		**CN**			**GH**		**CN**		
Treatment cycles	271		1,021		—	557		1,231		
Maternal age (yrs)	30.85 ± 2.68		30.70 ± 2.66		0.427	39.51 ± 3.18		39.37 ± 3.42		0.420
Maternal BMI (kg/m2)	22.86 ± 3.55		22.46 ± 3.63		0.103	23.00 ± 3.04		23.28 ± 3.60		0.117
Infertility duration (yrs)	3.67 ± 2.52		3.29 ± 2.48		0.030	4.77 ± 6.68		4.71 ± 4.60		0.805
Paternal age (yrs)	31.89 ± 3.41		32.17 ± 3.89		0.277	40.65 ± 5.38		40.55 ± 5.84		0.723
Primary infertility (%)	168	(61.99%)	627	(61.41%)	0.861	181	(32.50%)	416	(33.79%)	0.590
Nulliparous (%)	266	(98.15%)	981	(96.08%)	0.098	435	(78.10%)	975	(79.20%)	0.595
Main infertility cause (%)					0.069					0.555
Female	17	(6.27%)	101	(9.89%)		27	(4.85%)	75	(6.09%)	
Male	178	(65.68%)	604	(59.16%)		374	(67.15%)	818	(66.45%)	
Mixed	65	(23.99%)	247	(24.19%)		122	(21.90%)	277	(22.50%)	
Unexplained	11	(4.06%)	69	(6.76%)		34	(6.10%)	61	(4.96%)	
Basal hormone										
FSH (mIU/ml)	8.25 ± 4.34		7.64 ± 4.30		0.048	8.17 ± 3.78		8.27 ± 4.16		0.644
E2 (pmol/L)	208.99 ± 302.28		202.16 ± 213.19		0.689	228.30 ± 429.62		205.66 ± 207.65		0.154
LH (mIU/ml)	3.32 ± 2.23		3.46 ± 2.37		0.389	3.79 ± 3.02		3.68 ± 2.57		0.439
AMH (ng/ml)	0.96 ± 1.43		1.10 ± 1.27		0.098	0.76 ± 0.62		0.82 ± 0.74		0.055
AFC (%)					0.118					0.086
0–1	37	(13.65%)	96	(9.40%)		64	(11.49%)	105	(8.53%)	
2–3	63	(23.25%)	240	(23.51%)		162	(29.08%)	342	(27.78%)	
≥4	171	(63.10%)	685	(67.09%)		331	(59.43%)	784	(63.69%)	
Protocol (%)					< 0.001					< 0.001
LDR	23	(8.49%)	144	(14.10%)		42	(7.54%)	115	(9.34%)	
GnRH agonist long protocol	82	(30.26%)	328	(32.13%)		124	(22.26%)	311	(25.26%)	
Flare cycle	10	(3.69%)	26	(2.55%)		23	(4.13%)	32	(2.60%)	
GnRH antagonist protocol	132	(48.71%)	472	(46.23%)		293	(52.60%)	664	(53.94%)	
Micro stimulate protocol	24	(8.86%)	32	(3.13%)		75	(13.46%)	90	(7.31%)	
Natural cycle	0	(0.00%)	19	(1.86%)		0	(0.00%)	19	(1.54%)	
Total doses of gonadotropin (IU)	3,535 ± 1,289		3,492 ± 1,269		0.618	3,401 ± 1,382		3,577 ± 1,280		0.009
Endometrial thickness (mm)	10.57 ± 2.19		10.71 ± 1.79		0.342	10.07 ± 1.98		10.10 ± 2.00		0.814
No. of cycles canceled before oocyte retrieval (%)	7	(2.58%)	22	(2.15%)	0.672	22	(3.95%)	40	(3.25%)	0.453

*GH, growth hormone; CN, control; BMI, body mass index; FSH, follicle-stimulating hormone; LH, luteinizing hormone; E2, estradiol; AMH, Anti-Mullerian hormone; AFC, Antral follicle count; LDR, Long down-regulation*.

The clinical outcomes are depicted in Table 3. No statistically significant differences were found in the day 3 embryo available rate (52.31 vs. 54.70%; *p* = 0.131), the number of transferred embryos (1.66 ± 0.61 vs. 1.69 ± 0.60; *p* = 0.445), cycles reaching embryo transfer [ET] (71.97 vs. 71.17%; *p* = 0.866), proportion of day 3 ET (94.21 vs. 94.51%; *p* = 0.871), IR (28.82 vs. 28.99%; *p* = 0.953), MR(5.33 vs. 12.54%; *p* = 0.076), CPR (40.98 vs. 41.65%; *p* = 0.870), and LBR (37.70 vs. 34.54%; *p* = 0.426) between the two groups. In subgroup analysis, the LBR was similar between the GH and control groups with single embryo transfer stratified by days 3 and 5 transfers (D3, 30.00 vs. 18.99%; *p* = 0.215 and D5, 10.00 vs. 15.38%; *p* = 1.000). The trend was the same in double embryo transfers. We conducted logistic regression to adjust bias between the two groups. GH adjuvant therapy may not improve the LBR in young expected PORs (OR, 1.27; 95% CI, 0.88–1.85; *p* = 0.203; Table 4), while GH may not reduce the miscarriage rate in young expected PORs (OR, 0.37; 95% CI, 0.11–1.24; *p* = 0.108; Table 5).

**Table 3:** Clinical outcomes.

	**Maternal age** ** <35**					**Maternal age** **≥35**				
	**GH**		**CN**		***P*-value**	**GH**		**CN**		***P*-value**
No. of oocyte retrieval cycles (%)	264		999		–	535		1,191		–
No. of oocytes collected	6.56	± 4.69	7.66	± 5.09	0.001	5.55	± 3.93	5.40	± 4.43	0.513
No. of cleavage oocytes	1,212		5,395			2,095		4,612		
Day 3 embryo available rate	634	(52.31%)	2,951	(54.70%)	0.131	1,059	(50.55%)	2,353	(51.02%)	0.721
Cycles reaching ET/OR (%)	190	(71.97%)	711	(71.17%)	0.866	342	(63.93%)	778	(65.32%)	0.574
Day 3 ET	179	(94.21%)	672	(94.51%)	0.871	324	(94.74%)	716	(92.03%)	0.105
Day 5 ET	11	(5.79%)	39	(5.49%)		18	(5.26%)	62	(7.97%)	
No. of transferred embryo	1.66	± 0.61	1.69	± 0.60	0.445	1.54	± 0.70	1.59	± 0.64	0.221
Clinical pregnancy rate/ET (%)	75	(40.98%)	287	(41.65%)	0.824	78	(24.38%)	189	(25.61%)	0.591
Total No. of transplant embryos	340		1,304		–	595		1,344		–
Implantation rate/ET (%)	98	(28.82%)	378	(28.99%)	0.953	90	(15.13%)	222	(16.52%)	0.442
Miscarriage rate/CP (%)	4	(5.33%)	36	(12.54%)	0.076	20	(25.64%)	44	(23.28%)	0.681
Live birth rate/ET (%)	69	(37.70%)	238	(34.54%)	0.426	57	(17.81%)	132	(17.89%)	0.977
Day 3 ET	67	(37.43%)	232	(34.52%)	0.469	55	(16.98%)	121	(16.90%)	0.976
SET	9/30	(30.00%)	15/79	(18.99%)	0.215	7/71	(9.86%)	13/150	(8.67%)	0.773
DET	58/149	(38.93%)	217/593	(36.59%)	0.598	48/253	(18.97%)	108/566	(19.08%)	0.971
Day 5 ET	2	(18.18%)	6	(15.38%)	1.000	2	(11.11%)	11	(17.74%)	0.364
SET	1/10	(10.00%)	6/39	(15.38%)	1.000	2/18	(11.11%)	11/62	(17.74%)	0.722
DET	1/1	(100%)	–		–	–		–		–

*GH, growth hormone, CN, control, OR, oocyte retrieved, ET, embryo transfer, CP, clinical pregnancy, SET, single embryo transfer, DET, double embryo transfer*.

**Table 4:** Logistic regression for live births, while adjusting for confounders.

	**Maternal age** **<35 years**		**Maternal age** **≥35 years**	
	***P*-value**	**OR 95%C.I**	***P*-value**	**OR 95%C.I**
GH	0.203	1.27 (0.88–1.85)	0.342	1.20 (0.82–1.76)
Maternal age	0.548	1.02 (0.96–1.08)	0.001	0.76 (0.71–0.82)
Maternal BMI	0.267	0.98 (0.93–1.02)	0.777	1.01 (0.96–1.06)
Infertility duration	0.627	0.98 (0.92–1.05)	0.468	0.98 (0.94–1.03)
Primary infertility	0.162	0.80 (0.58–1.10)	0.295	0.81 (0.55–1.20)
Nulliparous	0.396	0.69 (0.29–1.63)	0.592	0.87 (0.51–1.47)
**Main infertility cause**				
Male	0.321		0.121	
Female	0.817	0.94 (0.56–1.58)	0.319	0.72 (0.38–1.37)
Mixed	0.386	1.29 (0.73–2.28)	0.164	0.61 (0.30–1.23)
Unexplained	0.487	1.30 (0.62–2.76)	0.468	01.39 (0.57–3.35)
AMH	0.453	0.94 (0.81–1.10)	0.591	0.93 (0.70–1.23)
AFC	0.129	0.96 (0.92–1.01)	0.703	1.01 (0.96–1.07)
**Protocol**				
GnRH agonist long protocol	0.242		0.331	
LDR	0.207	1.35 (0.85–2.15)	0.248	1.38 (0.80–2.40)
GnRH antagonist	0.19	0.79 (0.56–1.12)	0.266	0.79 (0.53–1.19)
Flare cycle	0.631	1.28 (0.47–3.51)	0.432	1.55 (0.52–4.60)
Micro stimulate	0.524	1.34 (0.54–3.31)	0.449	0.65 (0.21–2.00)
Natural cycle	0.999	0	1.000	0
No. of collected oocytes	0.001	1.08 (1.03–1.12)	0.987	1.00 (0.95–1.06)
No. of transferred embryos	0.038	1.76 (1.03–1.12)	0.014	2.04 (1.16–3.58)
Day 3/Day 5	0.145	0.49 (0.19–1.28)	0.522	1.33 (0.55–3.19)
Constant	0.999	0.01	0.999	421.6

*GH, growth hormone; BMI, body mass index; AMH, Anti-Mullerian hormone; AFC, Antral follicle count; OR, odd risk; CI, confidence interval; LDR, Long down-regulation*.

**Table 5:** Logistic regression for miscarriage, while adjusting for confounders.

	**Maternal age** **<** **35 years**		**Maternal age** **≥** **35 years**	
	***P*-value**	**OR 95%C.I**	***P*-value**	**OR 95%C.I**
GH	0.108	0.37 (0.11–1.24)	0.813	0.91 (0.43–1.93)
Maternal age	0.040	0.86 (0.75–0.99)	0.000	1.42 (1.22–1.66)
Maternal BMI	0.600	1.03 (0.92–1.16)	0.998	1.00 (0.91–1.10)
Infertility duration	0.581	0.95 (0.80–1.13)	0.478	0.97 (0.88–1.06
Primary infertility	0.360	1.46 (0.65–3.32)	0.878	1.06 (0.48–2.34)
Nulliparous	0.647	1.45 (0.30–7.12)	0.561	0.74 (0.26–2.07)
**Main infertility cause**				
Male	0.461		0.293	
Female	0.413	1.80 (0.44–7.34)	0.998	347498568.20 (0.00–)
Mixed	0.776	0.79 (0.15–4.02)	0.998	652989926.33 (0.00–)
Unexplained	0.660	1.58 (0.21–11.93)	0.998	215804921.68 (0.00–)
AMH	0.687	1.11 (0.66–1.86)	0.694	1.11 (0.67–1.83)
AFC	0.225	1.07 (0.96–1.20)	0.094	0.89 (0.77–1.02)
**Protocol**				
GnRH agonist long protocol	0.427		0.556	
LDR	0.512	0.70 (0.23–2.06)	0.422	1.50 (0.56–4.04)
GnRH antagonist	0.051	0.39 (0.15–1.00)	0.333	0.67 (0.29–1.52)
Flare cycle	0.999	0.00 (0.00–)	0.915	1.09 (0.21–5.60)
Micro stimulate	0.999	0.00 (0.00–)	0.427	0.40 (0.04–3.79)
No. of collected oocytes	0.001	0.78 (0.67–0.90)	0.688	0.98 (0.88–1.09)
No. of transferred embryos	0.091	0.36 (0.11–1.18)	0.335	0.59 (0.20–1.74)
Day3/Day5	0.502	1.79 (0.33–9.74)	0.851	0.86 (0.17–4.40)
Constant	1.000	0.10	0.994	0.00

*GH, growth hormone; BMI, body mass index; AMH, Anti-Mullerian hormone; AFC, Antral follicle count; OR, odd risk; CI, confidence interval; LDR, Long down-regulation*.

### Clinical Outcomes in PG 4

A total of 1,788 women were recruited to the PG4 group. In this group, 557 and 1,231 women were assigned to the GH adjuvant therapy and control groups, respectively. As shown in Table 2, there was no statistically significant difference in the baseline characteristics of patients and ovarian stimulation between GH treatment and control groups, except the protocol used for ovarian stimulation (*p* < 0.001) and dose of gonadotrophin (3,401 ± 1,382 IU vs. 3,577 ± 1,280 IU; *p* = 0.009).

The clinical outcomes are shown in Table 3. Similar to the PG3 group, no statistically significant differences were found in the day 3 embryo available rate (50.55 vs. 51.02%; *p* = 0.721) the number of transferred embryos (1.54 ± 0.70 vs. 1.59 ± 0.64; *p* = 0.221), cycles reaching ET (63.93 vs. 65.32%; *p* = 0.574), proportion of day 3 ET (94.74 vs. 92.03%; *p* = 0.105), IR (15.13 vs. 16.52%; *p* = 0.442), CPR (24.38 vs. 25.61%; *p* = 0.671), MR (25.64 vs. 23.28%; *p* = 0.681), and LBR (17.81 vs. 17.89%; *p* = 0.977) between the two groups. In subgroup analysis, the LBR was similar between the GH and control groups with single embryo transfer stratified by days 3 and 5 transfers (D3, 9.86 vs. 8.67%; *p* = 0.773 and D5, 11.11 vs. 17.74%; *p* = 0.722). The trend was the same in double embryo transfers. Logistic regression indicated that GH adjuvant therapy may not improve the LBR in old expected PORs (OR, 1.20; 95% CI, 0.82–1.76; *p* = 0.342; Table 4), while GH may not reduce the miscarriage rate in old expected PORs (OR, 0.91; 95% CI, 0.43–1.93; *p* = 0.813; Table 5).

### Subgroup Analysis in PG3 and PG4

The participants were stratified into three subgroups based on the ovarian stimulation protocol. The baseline characteristics of patients in the subgroups that underwent the LDR, GnRH-a long and antagonist protocols are shown in Supplementary Tables 1–3. As shown in Supplementary Tables 1, 2, there were no significant differences in the number of oocytes collected between GH treatment and control. The day 3 embryo available rate after GH treatment in young PORs who underwent LDR was significantly higher than the control group (63.11 vs. 49.35%; *p* = 0.004). As shown in Supplementary Table 3, the number of oocytes collected following GH treatment was significantly lower than the control group in subgroup that underwent the antagonist protocol (<35 years [5.92 ± 4.35 vs. 7.04 ± 4.83; *p* = 0.019]); however, the day 3 embryo available rate was not significantly different between the two groups in the subgroup that underwent the antagonist protocol (53.43 vs. 57.32; *p* = 0.101). Finally, LBR was comparable between GH treatment and control in GnRH antagonist (<35 years [35.19 vs. 28.45%; *p* = 0.183]; ≥35 years [12.96 vs. 14.03%; *p* = 0.707]), GnRH-a long protocol (<35 years [33.33 vs. 36.99%; *p* = 0.597]; ≥35 years [17.44 vs. 20.28%; *p* = 0.574]) and LDR (<35 years [58.82 vs. 41.90%; *p* = 0.193]; ≥35 years [43.33 vs. 25.30%; *p* = 0.065]; Table 6).

**Table 6:** Clinical outcomes based on ovarian stimulation protocol.

	**Maternal age** ** <35**					**Maternal age** **≥35**				
	**GH**		**CN**		***P*-value**	**GH**		**CN**		***P*-value**
**LDR**										
No. of oocytes collected	8.41	± 4.49	8.45	± 4.71	0.969	8.45	± 4.99	7.43	± 5.38	0.287
No. of cleavage oocytes	122		845			243		592		
Day 3 embryo available rate	77	(63.11%)	417	(49.35%)	0.004	109	(44.86%)	272	(45.95%)	0.774
Cycles reaching ET	17		105		–	30		83		–
Clinical pregnancy rate/ET (%)	10	(58.82%)	56	(53.33%)	0.673	14	(48.28%)	31	(38.27%)	0.347
Miscarriage rate/CP (%)	0	(0%)	8	(14.29%)	0.342	2	(14.29%)	9	(29.03%)	0.458
Live birth rate/ET (%)	10	(58.82%)	44	(41.90%)	0.193	12	(43.33%)	21	(25.30%)	0.065
**GnRH agonist long protocol**										
No. of oocytes collected	8.21	± 5.18	9.08	± 5.10	0.167	7.38	± 4.66	6.87	± 4.59	0.298
No. of cleavage oocytes	461		2,105			656		1,537		
Day 3 embryo available rate	228	(49.46%)	1,134	(53.87%)	0.085	324	(49.39%)	788	(51.27%)	0.420
Cycles reaching ET	60		246		–	86		217		–
Clinical pregnancy rate/ET (%)	25	(41.67%)	106	(43.09%)	0.842	20	(23.26%)	61	(28.11%)	0.389
Miscarriage rate/CP (%)	4	(16%)	13	(12.26%)	0.740	5	(25%)	14	(22.95%)	1.000
Live birth rate/ET (%)	20	(33.33%)	91	(36.99%)	0.597	15	(17.44%)	44	(20.28%)	0.574
**GnRH antagonist protocol**										
No. of oocytes collected	5.92	± 4.35	7.04	± 4.83	0.019	5.07	± 3.14	4.88	± 4.10	0.478
No. of cleavage oocytes	539		2,303			1,017		2,246		
Day 3 embryo available rate	288	(53.43%)	1,320	(57.32%)	0.101	538	(52.90%)	1,180	(52.54%)	0.848
Cycles reaching ET	108		341		–	538		1180		–
Clinical pregnancy rate/ET (%)	39	(36.11%)	119	(34.90%)	0.818	40	(18.52%)	90	(20.04%)	0.642
Miscarriage rate/CP (%)	0	(0%)	15	(12.61%)	0.023	12	(30%)	18	(20%)	0.212
Live birth rate/ET (%)	38	(35.19%)	97	(28.45%)	0.183	28	(12.96%)	63	(14.03%)	0.707

*GH, growth hormone; CN, control; ET, embryo transfer; CP, clinical pregnancy; LDR, Long down-regulation*.

## Discussions

### Main Findings

This study demonstrated that GH adjuvant therapy may not increase the chance of achieving live birth in expected PORs, when adjusted for confounding factors and conducting a sub-analysis with embryo transfer number (single vs. double) and different protocols; however, GH treatment significantly improved the day 3 embryo quality in young PORs who underwent LDR.

### Strengths and Limitations

The strengths of this study were as follows. First, the patients included in this study fulfilled the POSEIDON criteria. POSEIDON was proposed to reduce the population heterogeneity of Bologna (29). Second, the sample size of this study was relatively large, while the duration of the study was only 18 months; thus, this study could reduce the bias of IVF/ICSI protocols at the same time. The study was mainly limited by the retrospective nature. GH supplementation may be selectively adopted to treat difficult PORs with a relatively low AFC and longer duration of infertility in the PG3 group (<35 years). In addition, this study did not include outcomes of cryopreserved embryos, thus the effectiveness of GH treatment in terms of a cumulative LBR may be underestimated (30).

### Interpretation of Data

The rationale for add-on of GH to treat PORs was due to stimulation of IGF1, which has been reported to have synergistic effects with FSH on follicular development in an animal study (13). Moreover, GH treatment in older PORs increased the density of granulosa FSH and LH receptors (14). Two meta-analyses with few non-Bologna patients demonstrated statistically significant differences in CPR and LBR favoring the use of GH (24, 31). In contrast, a relatively large RCT, including 141 Bologna PORs, suggested that there was no statistically significant difference in CPR and LBR between the GH treatment and control groups (21). The other RCT conducted by LIGHT investigators, including 130 patients with at least one cycle of a poor response to COH, showed that the GH and control groups had a similar probability of achieving live births (32). Taken together, the discrepant findings may be related to different criteria employed for defining PORs (28). The heterogeneity of patients is associated with different underlying etiologies of PORs and may result in a variety of GH intervention effects. Based on clinical trials, there is no robust evidence to support widespread use of GH in treating PORs to date.

In this study, the distribution of AFC in the GH supplement group was skewed to an extremely low number (i.e., 0–1) in the PG3 group, but the proportion of AFC distribution did not reach a statistically significant difference between the two groups. GH treatment may be preferentially used in some extremely difficult PORs in our clinics, especially for young PORs (<35 years of age). The duration of infertility and AFC bias was reflected by the lower number of oocytes collected after GH treatment in young PORs (PG3 group); however, the day 3 embryo available rate was comparable in young PORs (PG3 group). In contrast, there was no bias with respect to duration of infertility and AFC in old PORs (PG4 group); the number of oocytes collected and the day 3 embryo available rate were comparable in old PORs. In fact, the physician considered selecting young PORs to undergo GH treatment. The reduced total dose of gonadotrophins may be due to a higher proportion of patients undergoing the micro-stimulation protocol in the GH treatment group among PG4 patients. Nevertheless, these results did not favor GH usage in improving the CPR and LBR in expected PORs, the finding was in agreement with two recently published RCTs (21, 32). Moreover, recently a review suggested no benefit of increased LBR for PORs (33). Of note, there were significant difference in the COH protocol and number of oocytes collected. It has been reported that different stimulation protocols (agonist vs. antagonist) (34) and the number of oocytes collected (35, 36) may cause bias on the LBR. Hence, duration of infertility, the number of AFC, the number of oocytes collected, and COH protocol were adjusted in logistic regression along with other potential confounding factors. Finally, the results of logistic regression indicated that GH addition was not significantly associated with the LBR.

In previous studies, efficacy of GH treatment on CPR and LBR was investigated in PORs with different protocols; including antagonist (21, 23, 32), GnRH-a long protocol (22), mild stimulation protocol (37), the result was not consistent in these studies. Moreover, a prospective randomized trial suggested that the GnRH-a long protocol was superior to the other three protocols, such as GnRH-a short, mini-flare, and antagonist protocols, regarding the number of oocytes retrieved and fertilized, but the CPR difference was not statistically significant (38). Thus, it is necessary to break out PORs with different protocols. Consequently, PORs were further stratified based on the COH protocol to investigate which subgroup was optimal in terms of LBR, and while eliminating major bias in this study. The number of oocytes collected was not different between the two groups in the LDR and GnRH-a long protocols however, the day 3 embryo available rate was significantly increased after GH treatment in young PORs with LDR. Nevertheless, a similar CPR and LBR were observed in PORs with LDR and GnRH-a long protocols. Of note, the miscarriage rate was significantly reduced after GH usage in young PORs undergoing the antagonist protocol, but the LBR was still comparable between the GH treatment and control group in PORs undergoing the antagonist protocol. The risk of pregnancy loss was influenced by several factors, including patient's characteristics and embryo quality (39). There was no significant difference in patient characteristics, except for the AMH level. Because a lower ovarian reserve existed in the GH treatment group, the number of oocytes collected was significantly lower in the GH treatment group; however, the number of available embryos between the two groups was comparable. Therefore, the reduced miscarriage rate in PORs who underwent the antagonist protocol was likely to be associated with improved embryo quality after GH treatment (39). The same effect on improving embryo quality was observed in young PORs who underwent LDR after GH treatment. The lower trend in miscarriages was weak after GH treatment with the antagonist protocol because the total number of miscarriage events was very small (*n* = 15). Finally, logistic regression indicated that GH treatment was not associated with a risk of pregnancy loss in young and old PORs with a large sample size.

## Conclusion

In conclusion, this study with expected PORs classified by the POSEIDON criteria demonstrated that growth hormone adjuvant therapy may not increase the probability of achieving a live birth in expected PORs whether single or double embryo transfer; however, a positive effect on embryo quality improvement was observed on sub-analysis.

## Statement

Poor ovarian responder (POR) was a therapeutic challenge in assisted reproduction treatment, mainly characterized by low ovarian reserve or poor response in previous ovarian stimulation. They were estimated to occur in 5.6–35.1% of patients, depending on the definition of POR. Growth hormone (GH) has been used for many years to increase the ovarian response in clinical practice. Previous literatures have found the benefit of GH usage with reduction in duration of ovarian stimulation, with a greater number of oocyte retrieved, and improvement in early clinical parameters. However, there is a lack of a clear definition of POR and participation heterogeneity in previous study. Until now, no benefit of GH usage with increased chance of live birth was determined. In this study, we set out to investigate the efficacy of GH adjuvant therapy in expected PORs based on POSEIDON classification. The results showed that growth hormone adjuvant therapy may not increase the probability of achieving live birth.

## Data Availability Statement

The datasets generated for this study are available on request to the corresponding author.

## Ethics Statement

The studies involving human participants were reviewed and approved by Ethics Committee of Reproductive Medicine, Peking University Third Hospital. Written informed consent for participation was not required for this study in accordance with the national legislation and the institutional requirements.

## Author Contributions

JQ: conceived, designed the study, and revised the manuscript. PL and RL: coordinated data collection. JZ and YW: analyzed the data and drafted the manuscript. LC: statistical analysis.

### Conflict of Interest

The authors declare that the research was conducted in the absence of any commercial or financial relationships that could be construed as a potential conflict of interest.
