# Supplementary material for: Growth Hormone Supplementation May Not Improve Live Birth Rate in Poor Responders
Source: Front Endocrinol (Lausanne). 2020 Jan 23;11:1. doi: 10.3389/fendo.2020.00001 (PMC6990136; doi:10.3389/fendo.2020.00001)
Supplement: Supplementary file 1 [file Data_Sheet_1.docx]

**Supplementary Table 1. Basal characteristics of patients in the subgroup undergoing the LDR**

|  | Maternal age <35 | | | | | | | | | Maternal age ≥35 | | | | | | | | |
| --- | --- | --- | --- | --- | --- | --- | --- | --- | --- | --- | --- | --- | --- | --- | --- | --- | --- | --- |
|  | GH | | | | CN | | | | P-value |  |  | GH |  |  |  | CN |  | P-value |
| Treatment cycles |  |  | 23 |  |  |  | 144 |  |  |  |  | 42 |  |  |  | 115 |  |  |
| Maternal age (yrs) | 31.43±2.23 | | | | 30.77±2.76 | | | | 0.274 | 38.07±2.61 | | | | 37.70±2.79 | | | | 0.449 |
| Maternal BMI | 22.22±2.99 | | | | 22.61±3.61 | | | | 0.624 | 22.60±2.99 | | | | 23.30±3.84 | | | | 0.283 |
| Infertility duration (yrs) | 3.22±1.65 | | | | 3.32±2.60 | | | | 0.850 | 4.85±4.74 | | | | 4.53±4.12 | | | | 0.683 |
| Paternal age (yrs) | 31.48±2.87 | | | | 32.33±3.78 | | | | 0.301 | 39.88±4.12 | | | | 38.61±5.07 | | | | 0.146 |
| Primary infertility |  | 18 | (78.26%) |  |  | 89 | (61.81%) |  | 0.127 | 16 | ( | (38.10%) | ) | 49 |  | (42.61%) |  | 0.611 |
| Nulliparous |  | 23 | (100%) |  |  | 140 | (97.22%) |  | 1.000 | 39 | ( | (92.86%) | ) | 103 |  | (89.57%) |  | 0.761 |
| Main infertility cause |  |  |  |  |  |  |  |  | 0.773 |  |  |  |  |  |  |  |  | 0.295 |
| Female |  | 15 | (65.22%) |  |  | 88 | (61.11%) |  | |  | 27 | (64.29%) |  |  | 69 | (60.00%) |  | |
| Male |  | 2 | (8.70%) |  |  | 10 | (6.94%) |  | |  | 2 | (4.76%) |  |  | 5 | (4.35%) |  | |
| Mixed |  | 6 | (26.09%) |  |  | 40 | (27.78%) |  | |  | 7 | (16.67%) |  |  | 33 | (28.70%) |  | |
| Unexplained |  | 0 | (0.00%) |  |  | 6 | (4.17%) |  | |  | 6 | (14.29%) |  |  | 8 | (6.96%) |  | |
| AMH | 1.17±0.82 | | | | 1.43±1.49 | | | | 0.420 | 1.09±0.75 | | | | 1.07±0.82 | | | | 0.916 |
| AFC |  |  |  |  |  |  |  |  | 0.211 |  |  |  |  |  |  |  |  | 0.773 |
| 0~1 |  | 1 | (4.35%) |  |  | 19 | (13.19%) |  | |  | 3 | (7.14%) |  |  | 12 | (10.43%) |  | |
| 2~3 |  | 4 | (17.39%) |  |  | 39 | (27.08%) |  | |  | 8 | (19.05%) |  |  | 24 | (20.87%) |  | |
| ≥4 |  | 18 | (78.26%) |  |  | 86 | (59.72%) |  | |  | 31 | (73.81%) |  |  | 79 | (68.70%) |  | |

LDR: Long down-regulation

GH: growth hormone

CN: control

BMI: body mass index

AMH: Anti-Mullerian hormone

AFC: Antral follicle count

**Supplementary Table 2. Basal characteristics of patients in the subgroup undergoing the GnRH agonist long protocol**

|  | Maternal age <35 | | | | | | | | | Maternal age ≥35 | | | | | | | | |
| --- | --- | --- | --- | --- | --- | --- | --- | --- | --- | --- | --- | --- | --- | --- | --- | --- | --- | --- |
|  | GH | | | | CN | | | | P-value |  |  | GH |  |  |  | CN |  | P-value |
| Treatment cycles |  |  | 82 |  |  |  | 328 |  |  |  |  | 124 |  |  |  | 311 |  |  |
| Maternal age (yrs) | 30.80±2.71 | | | | 30.70±2.67 | | | | 0.747 | 39.15±3.05 | | | | 38.53±3.00 | | | | 0.053 |
| Maternal BMI | 22.12±3.25 | | | | 22.04±3.53 | | | | 0.853 | 23.17±3.06 | | | | 22.92±3.53 | | | | 0.499 |
| Infertility duration (yrs) | 3.29±2.52 | | | | 3.00±2.13 | | | | 0.298 | 4.75±4.40 | | | | 4.95±4.57 | | | | 0.678 |
| Paternal age (yrs) | 31.85±3.52 | | | | 32.08±3.99 | | | | 0.644 | 40.44±4.93 | | | | 39.75±5.65 | | | | 0.236 |
| Primary infertility |  | 45 | (54.88%) |  |  | 203 | (61.89%) |  | 0.245 |  | 31 | (25.00%) |  |  | 108 | (34.73%) |  | 0.050 |
| Nulliparous |  | 81 | (98.78%) |  |  | 313 | (95.43%) |  | 0.213 |  | 94 | (75.81%) |  |  | 265 | (85.21%) |  | 0.020 |
| Main infertility cause |  |  |  |  |  |  |  |  | 0.056 |  |  |  |  |  |  |  |  | 0.977 |
| Female |  | 55 | (67.07%) |  |  | 185 | (56.40%) |  | |  | 78 | (62.90%) |  |  | 193 | (62.06%) |  | |
| Male |  | 5 | (6.10%) |  |  | 59 | (17.99%) |  | |  | 9 | (7.26%) |  |  | 24 | (7.72%) |  | |
| Mixed |  | 18 | (21.95%) |  |  | 64 | (19.51%) |  | |  | 30 | (24.19%) |  |  | 79 | (25.40%) |  | |
| Unexplained |  | 4 | (4.88%) |  |  | 20 | (6.10%) |  | |  | 7 | (5.65%) |  |  | 15 | (4.82%) |  | |
| AMH | 1.29±2.35 | | | | 1.25±1.44 | | | | 0.847 | 0.98±0.63 | | | | 0.93±0.78 | | | | 0.592 |
| AFC |  |  |  |  |  |  |  |  | 0.708 |  |  |  |  |  |  |  |  | 0.024 |
| 0~1 |  | 7 | (8.54%) |  |  | 28 | (8.54%) |  | |  | 10 | (8.06%) |  |  | 20 | (6.43%) |  | |
| 2~3 |  | 18 | (21.95%) |  |  | 59 | (17.99%) |  | |  | 37 | (29.84%) |  |  | 58 | (18.65%) |  | |
| ≥4 |  | 57 | (69.51%) |  |  | 241 | (73.48%) |  | |  | 77 | (62.10%) |  |  | 233 | (74.92%) |  | |

GH: growth hormone

CN: control

BMI: body mass index

AMH: Anti-Mullerian hormone

AFC: Antral follicle count

**Supplementary Table 3. Basal characteristics of patients in the subgroup undergoing the GnRH antagonist protocol**

|  | Maternal age <35 | | | | | | | | | Maternal age ≥35 | | | | | | | | |
| --- | --- | --- | --- | --- | --- | --- | --- | --- | --- | --- | --- | --- | --- | --- | --- | --- | --- | --- |
|  | GH | | | | CN | | | | P-value |  |  | GH |  |  |  | CN |  | P-value |
| Treatment cycles |  |  | 132 |  |  |  | 472 |  |  |  |  | 293 |  |  |  | 664 |  |  |
| Maternal age (yrs) | 30.73±2.78 | | | | 30.67±2.68 | | | | 0.828 | 39.55±3.10 | | | | 39.60±3.37 | | | | 0.840 |
| Maternal BMI | 23.20±3.69 | | | | 22.72±3.75 | | | | 0.192 | 22.84±2.94 | | | | 23.55±3.70 | | | | 0.004 |
| Infertility duration (yrs) | 3.77±2.44 | | | | 3.45±2.66 | | | | 0.218 | 4.50±4.50 | | | | 4.53±4.50 | | | | 0.915 |
| Paternal age (yrs) | 31.96±3.52 | | | | 32.17±3.97 | | | | 0.580 | 40.76±5.72 | | | | 40.73±5.83 | | | | 0.938 |
| Primary infertility |  | 81 | (61.36%) |  |  | 286 | (60.59%) |  | 0.873 |  | 97 | (33.11%) |  |  | 209 | (31.48%) |  | 0.618 |
| Nulliparous |  | 128 | (96.97%) |  |  | 452 | (95.76%) |  | 0.530 |  | 223 | (76.11%) |  |  | 502 | (75.60%) |  | 0.866 |
| Main infertility cause |  |  |  |  |  |  |  |  | 0.354 |  |  |  |  |  |  |  |  | 0.907 |
| Female |  | 86 | (65.15%) |  |  | 285 | (60.38%) |  | |  | 200 | (68.26%) |  |  | 460 | (69.28%) |  | |
| Male |  | 9 | (6.82%) |  |  | 32 | (6.78%) |  | |  | 15 | (5.12%) |  |  | 34 | (5.12%) |  | |
| Mixed |  | 33 | (25.00%) |  |  | 121 | (25.64%) |  | |  | 61 | (20.82%) |  |  | 139 | (20.93%) |  | |
| Unexplained |  | 4 | (3.03%) |  |  | 34 | (7.20%) |  | |  | 17 | (5.80%) |  |  | 31 | (4.67%) |  | |
| AMH | 0.78±0.64 | | | | 1.00±1.11 | | | | 0.028 | 0.71±0.60 | | | | 0.81±0.73 | | | | 0.050 |
| AFC |  |  |  |  |  |  |  |  | 0.197 |  |  |  |  |  |  |  |  | 0.500 |
| 0~1 |  | 17 | (12.88%) |  |  | 38 | (8.05%) |  | |  | 28 | (9.56%) |  |  | 49 | (7.38%) |  | |
| 2~3 |  | 32 | (24.24%) |  |  | 109 | (23.09%) |  | |  | 78 | (26.62%) |  |  | 187 | (28.16%) |  | |
| ≥4 |  | 83 | (62.88%) |  |  | 325 | (68.86%) |  | |  | 187 | (63.82%) |  |  | 428 | (64.46%) |  | |

GH: growth hormone

CN: control

BMI: body mass index

AMH: Anti-Mullerian hormone

AFC: Antral follicle count
